# Elimination of ‘the Glasgow effect’ in levels of dental caries in Scotland’s five-year-old children: 10 cross-sectional surveys (1994–2012)

**DOI:** 10.1186/s12889-015-1492-0

**Published:** 2015-03-04

**Authors:** Yvonne I Blair, Alex D McMahon, Wendy Gnich, David I Conway, Lorna MD Macpherson

**Affiliations:** Oral Health Directorate, NHS Greater Glasgow & Clyde, Glasgow Dental Hospital, 378 Sauchiehall St, Glasgow, G2 3JZ Scotland UK; Community Oral Health Section, University of Glasgow Dental School, 378 Sauchiehall St, Glasgow, G2 3JZ Scotland UK

**Keywords:** ‘Glasgow Effect’, Socio-economic inequalities, Deprivation, Dental caries, Child health, Epidemiology

## Abstract

**Background:**

Socioeconomic inequalities in health within Glasgow, Scotland, are among the widest in the world. This is largely attributed to socio-economic conditions. The ‘Glasgow Effect’ labels the finding that the high prevalence of some diseases cannot be fully explained by a conventional area-based socio-economic metric. This study aimed to investigate whether differences in dental caries between Glasgow’s resident children and those in the Rest of Scotland could be explained by this metric and whether differences were of fixed magnitude, over time.

**Methods:**

Scotland’s National Dental Inspection Programme (NDIP) cross-sectional data for five-year-old children in years: 1994, 1996, 1998, 2000, 2003, 2004, 2006, 2008, 2010, and 2012 (n = 92,564) were utilised. Endpoints were calculated from the mean decayed, missing and filled teeth score (d_3_mft) and percentage with obvious decay experience. Socioeconomic status was measured by DepCat, a Scottish area-based index. The Glasgow Effect was estimated by the odds-ratio (OR) of decay for Glasgow versus the Rest of Scotland adjusted by age, gender and DepCat. Inequalities were also assessed by the Significant Caries Index (SIC), SIC 10, and Scottish Caries Inequality Metric (SCIM 10).

**Results:**

Decay levels for deprived Glasgow children have reduced to be similar to those in the Rest of Scotland. In 1993, OR for d_3_mft > 0 for those living in the Glasgow area was 1.34(1.10, 1.64), p = 0.005. This reduced below unity in 2012, OR = 0.85(0.77, 0.93), p < 0.001. There were downward trends (p < 0.001) in absolute inequality measured by SIC and SIC 10 in each of the geographic areas. The SCIM 10 demonstrated further reductions in inequality across the population. The downward trends for all the inequality measures were larger for Glasgow than the Rest of Scotland.

**Conclusions:**

Over the interval, Glasgow has eliminated the earlier extra health inequalities. When comparing ‘like for like’ by socioeconomic status there is now no higher level of dental caries in the Greater Glasgow area.

## Background

Socioeconomic inequalities in life expectancy within the Glasgow conurbation, Scotland are among the widest in the world, with a 28 year difference between the poorest and healthiest neighbourhoods [1]. The incidence and prevalence rates for many chronic diseases in Glasgow are also among the highest in developed countries [1-3]. This poor health profile has been largely attributed to socio-economic conditions, since Glasgow has higher levels of deprivation than the Rest of Scotland and the UK [4]. However, in recent years a ‘Glasgow Effect’ has been identified and debated within the public health literature. The ‘Glasgow Effect’ labels the finding that the high prevalence of some diseases (and behaviours associated with ill health) observed in the city’s population cannot be fully explained by controlling for conventional socio-economic status (SES), using area and/or individual level measures, and that there may be ‘unknown drivers’ of poor health associated with living in the city [2,5-7]. Several explanations have been put forward for the ‘Glasgow Effect’ including: the sensitivity of measures of socio-economic status used; behavioural or biological variables; political and historical factors influencing residents’ sense of hopelessness and community coherence; and additional unknown factors associated with living in the Glasgow area [5-11]. It has been postulated that part of the explanation for the ‘Glasgow Effect’ on adult health outcomes is to be found in the adverse effects emanating from childhoods characterised by factors such as poverty and poor social status [12]. A recent paper by the Glasgow Centre for Population Health [13] has identified SES, psychological and biological associations with adverse physiological outcomes e.g. biological-aging, epigenetic effects and high levels of biomarkers of ill health.

Despite growing interest in the ‘Glasgow Effect’ there has been limited work investigating longitudinal changes or focusing on child health and health behaviours, with work published to-date suggesting that the excess burden associated with the effect had not previously been observed in the very young [10]. The prevalence of dental caries in young children living in the city affords an opportunity to investigate the effect within Glasgow’s child population over time. Dental caries is of interest as it shares many of the risk factors associated with other chronic diseases [14], and has had a high prevalence in the West of Scotland and in particular in the Glasgow area, with a strong association seen between socio-economic position and the prevalence and morbidity of the disease [15,16]. Moreover, population dental health outcomes can be measured from a young age, with collection of data from five-year-old children being undertaken on a routine basis across Scotland as part of a National Dental Inspection Programme (NDIP) [16]. Over the past 10–15 years, a number of child oral health improvement initiatives have been introduced within Glasgow and the rest of Scotland [17-20]. Improvements in the dental health of five-year-olds have been apparent over the past decade [16].

*Childsmile* is the national oral health improvement programme for Scotland established in 2006 [20]. It involves a blend of universal/population and targeted interventions. All children attending nursery/kindergarten educational provision are offered a daily supervised toothbrushing programme; and primary care dental services have been reoriented to provide prevention from birth. In addition fluoride varnish programmes are targeted to the most deprived (20%) nursery/kindergarten establishments, and additional Dental Health Support Worker involvement to support families in the most deprived areas based on area-based socioeconomic measurement [21].

The aims of this study were to investigate whether differences in dental caries between children residing in Glasgow and those residing elsewhere in Scotland could be fully explained by conventional area-based measures of socio-economic status or whether a ‘Glasgow Effect’ was observed and to determine whether the pattern changed over time as overall oral health has improved.

## Methods

This study comprised data analyses of datasets resulting from the National Dental Inspection Programme (NDIP) of Scotland. These datasets belong to Scotland’s 14 NHS Boards and are controlled on their behalf by the Scottish Dental Epidemiology Co-ordinating Committee [SDECC]. Scotland’s national caries datasets do not have open access. The SDECC granted access to the data for this study which utilised detailed NDIP data for five-year-old children (in the first academic year of primary education) gathered in the school setting in alternate years in Scotland. The NDIP data are generated as part of a Scottish Government oral health monitoring system and no further ethical approval is required for the analysis of these data. Legally, it is a statutory duty to inspect children at least twice. There is an option to opt-out of this programme, but opt-in consent is not applicable.

The specific academic periods covered in this study were: 1993/94, 1995/96, 1997/98, 1999/00, 2002/03, 2003/04, 2005/06, 2007/08, 2009/10, and 2011/12. For brevity, and based upon the year when the majority of inspections were carried out for each time point, the second year will be used for reporting.

The methods employed by NDIP have been described previously [16,22]. These population surveillance surveys, conducted by trained and calibrated dentist examiners, use the standardised diagnostic criteria of The British Association for the Study of Community Dentistry (BASCD) to measure dental caries at the level of visible penetration into the dentine layer of teeth or beyond (d_3_) [23]. The mean decayed, missing and filled teeth score i.e. the d_3_mft index and the prevalence of d_3_mft are the standard metrics reported in caries epidemiology [24]. Deprivation was measured by ‘DepCat’ (a composite area-based indicator of socioeconomic status). DepCat correlates consistently with Scotland’s morbidity and mortality data, it is long established [25], and it is derived from categories created from variables collected at the 2001 national decennial census at postcode sector level comprising proportions of: residents living in overcrowded households; unemployed males; persons in households headed by someone of low social class; and, persons who do not own a car [26]. Expert consensus suggests that DepCat (2001) should be Scotland’s area-based SES indicator of choice for retrospective analyses from 1991 onwards [27].

### Statistical analysis

Mean d_3_mft and the percentage of children with ‘no obvious decay experience’ (i.e. d_3_mft > 0) were calculated for two geographic subgroups residing in a) the area of former NHS Greater Glasgow Health Board (GGHB, n = 24,799 subjects) denoted simply as ‘Glasgow’ and b) the remainder of Scotland (n = 67,765 subjects) external to ‘Glasgow’ denoted as the ‘Rest of Scotland’, for each year of study. The DepCat (2001) score of each subject’s postcode of residence permitted analyses by area-based socioeconomic status. The geographic boundaries of the two study areas were stable over the study interval. The odds of decay were analysed using multivariable logistic regression. We present the odds-ratio of decay for Glasgow versus the Rest of Scotland, after adjustment for age, gender and DepCat. In this way the ‘Glasgow Effect’ can be directly captured by an independent odds-ratio. The attendant 95% confidence interval, a one degree of freedom Wald Chi-squared p-value, and a test of interaction between area (i.e. Glasgow/Rest of Scotland) and year of study were also calculated.

Additionally, the Significant Caries Index (SIC) [28], the SIC of the most deprived decile (SIC 10) [29] and the Scottish Caries Inequality Metric (SCIM 10) for Glasgow and the Rest of Scotland, were calculated separately for each year of study and geographic area. Their concurrent usefulness to describe population distributions of caries inequalities was described in a previous methodological paper on inequality metrics [22]. The SIC score was calculated by ranking d_3_mft scores of all individuals, irrespective of their DepCat using a 33% cutpoint. The SIC score is the mean d_3_mft of the highest third of the distribution, SIC 10 score uses the highest tenth of the distribution of d_3_mft, and SCIM 10 is an area under the curve measure calculated from the range of tenths of the distribution. The three inequality metrics were analysed for trend over year of study by using linear regression.

## Results

The mean age of the inspected children ranged from 5.2 in 1993 to 5.5 in 2012, and was 5.5 for all years combined both in Glasgow and the Rest of Scotland. There were equal numbers of boys and girls. Mean d_3_mft and the percentage of children with ‘no obvious decay experience’ (i.e. d_3_mft > 0) are tabulated in Table 1. In the Glasgow area mean d_3_mft reduced from 4.3 in 1993 to 1.6 in 2012 (a reduction of 2.7 or 63%), and reduced from 3.0 to 1.3 in the Rest of Scotland, respectively (a reduction of 1.7 or 57%). Similarly, in the Glasgow area the percentage of children with obvious decay reduced from 74% in 1993 to 36% in 2012 (an absolute reduction of 38% and relative reduction to 51%). In the Rest of Scotland the equivalent reduction was from 58% to 33%, an absolute reduction of 25% and a relative reduction to 43%.

**Table 1:** **Mean d**_**3**_ **mft, % obvious decay experience, Significant Caries Index (SIC), SIC of poorest decile (SIC 10) and Scottish Caries Inequality Metric (SCIM 10) for Glasgow and the Rest of Scotland per annum**

	**Glasgow (n = 24,799)**						**Rest of Scotland (n = 67,765)**					
**Year**	**Mean d**_**3**_ **mft (SD)**	**% decay**	**SIC**	**SIC 10**	**SCIM 10**	**n**	**Mean d**_**3**_ **mft (SD)**	**% decay**	**SIC**	**SIC 10**	**SCIM 10**	**n**
**1994**	4.3 (4.2)	74%	9.39	12.98	36.75	840	3.0 (3.8)	58%	7.59	11.55	24.22	4233
**1996**	3.6 (3.8)	67%	8.13	11.33	30.06	1648	2.8 (3.7)	56%	7.23	11.01	22.84	4587
**1998**	3.8 (4.0)	67%	8.69	12.13	32.31	1458	2.5 (3.4)	54%	6.50	10.38	19.77	5107
**2000**	3.6 (4.0)	66%	8.51	12.30	29.86	1080	2.4 (3.3)	52%	6.24	9.98	18.56	5686
**2003**	3.5 (3.8)	65%	8.02	11.21	29.16	2232	2.8 (3.6)	55%	7.07	10.63	22.20	7515
**2004**	3.1 (3.7)	59%	7.58	10.81	25.57	4263	2.2 (3.3)	47%	6.13	9.99	17.39	6693
**2006**	2.5 (3.4)	50%	6.62	10.11	19.70	3220	2.0 (3.1)	44%	5.69	9.42	15.60	7725
**2008**	2.1 (3.3)	45%	5.94	9.85	16.46	3422	1.8 (3.0)	41%	5.23	9.04	13.71	8645
**2010**	1.8 (3.0)	42%	5.28	9.07	13.97	3167	1.4 (2.7)	35%	4.28	8.35	10.23	8771
**2012**	1.6 (2.9)	36%	4.80	8.82	11.91	3469	1.3 (2.5)	33%	3.95	7.72	9.32	8803
Slope			−0.23	−0.21	−1.29				−0.18	−0.18	−0.76	
p=			<0.001	<0.001	<0.001				<0.001	<0.001	<0.001	

The inequality metrics (SIC, SIC 10, and SCIM 10) are tabulated for Glasgow and the Rest of Scotland by year of study in Table 1. In summary, SIC reduced from a mean d_3_mft of 9.39 to 4.80 in Glasgow (from 1993 to 2012) and reduced from 7.59 to 3.95 in the Rest of Scotland. The SIC 10 reduced from d_3_mft 12.98 to 8.82 in the Glasgow area and from 11.55 to 7.72 in the Rest of Scotland, respectively. The SCIM 10 measure, which is a summary across all the tenths of the distribution of mean d_3_mft, similarly reduced from 36.75 to 11.92 in the Glasgow area (a reduction of 24.84 or 68%) and from 24.22 to 9.32 in the Rest of Scotland (a reduction of 14.9 or 62%). The downward trend was significant in all cases (p < 0.001, for slopes) and it is clear that the downward trajectories are steeper for the Glasgow area than for the Rest of Scotland (Table 1).

**Figure 1:**
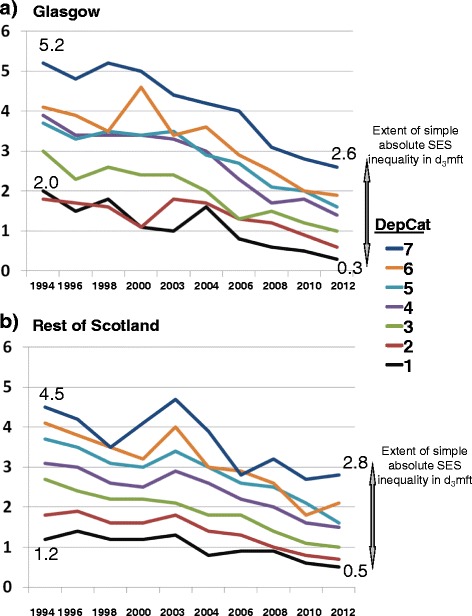
**Mean d**_**3**_ **mft by deprivation category (DepCat: 1 = least deprived, 7 = most deprived).**

**Figure 2:**
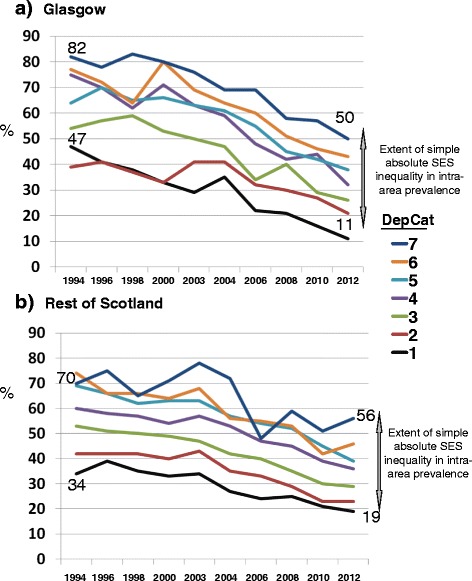
**% obvious decay experience by deprivation category (DepCat: 1 = least deprived, 7 = most deprived).**

Figures 1 and 2 demonstrate the direct associations of children’s DepCat with their mean d_3_mft scores and the prevalence of those with obvious decay experience (%d_3_mft > 0), respectively. In spite of the marked trends towards diminished overall mean d_3_mft scores and percentages with d_3_mft > 0 evident in the two geographic areas over time, at each cross-sectional study the gradients in morbidity and prevalence of caries by DepCat within the individual study years have persisted in both Glasgow and the Rest of Scotland. The ‘gaps’ illustrated in Figure 1 between the values for mean d_3_mft recorded for the children resident in DepCat 7 postcodes and those from DepCat 1 small areas in the respective geographic areas at individual study points are indicative of the magnitudes of simple absolute inequality in area-based caries burden experienced by the DepCat 7 children at each point, likewise for prevalence (Figure 2) of d_3_mft > 0. Furthermore, comparison of the separate values for mean d_3_mft for each DepCat in Figure 1 graphs (a) Glasgow and (b) the Rest of Scotland illustrates that caries morbidity was less severe for all children from each DepCat category in the Rest of Scotland than in Glasgow, at baseline. A similar comparison of the graphs (a) and (b) in Figure 2 shows that prevalence of d_3_mft > 0 in the Rest of Scotland also was lower within each DepCat category.

These figures demonstrate that the children living in more deprived areas had higher baseline levels of dental decay, and that these baseline levels were higher again in the Glasgow area. Over time the levels in children living in the more deprived areas of Glasgow have converged downwards to be similar to the deprived children in the Rest of Scotland.

**Figure 3:**
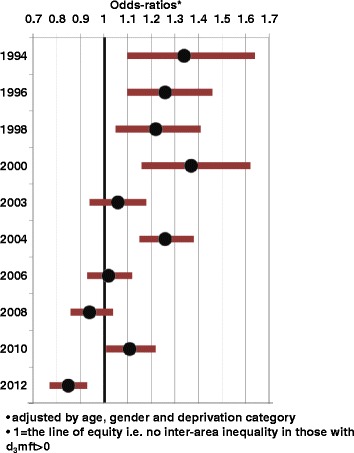
**% obvious decay experience [95%CI]: Glasgow versus Rest of Scotland, by year of study.**

The adjusted odd-ratios of decay (controlling for DepCat) for Glasgow versus the Rest of Scotland are illustrated in Figure 3 by year of study. In 1993 the odds-ratio for caries of the Glasgow area children was 1.34 (1.10, 1.64), p = 0.005. These odds-ratios have gradually reduced, with some oscillation, until 2012 when the odds-ratio became significantly lower than unity for the ‘Glasgow Effect’, i.e. 0.85 (0.77, 0.93), p < 0.001. A test of interaction between year of study and the Glasgow area variable was also significant, p < 0.001.

## Discussion

This study explored dental caries of Scotland’s five-year-olds over time. At all cross-sectional points when comparing Glasgow childrens’ caries prevalence and morbidity to those children resident in the Rest of Scotland, Glasgow children had a greater burden of disease [Table 1]. Notwithstanding this, there have been very dramatic improvements in mean number of teeth affected and in the prevalence of dental caries in both of these populations at a time when there is no evidence that strategies adopted by the NHS in other parts of the UK to decrease inequalities are resulting in similar effects [30]. Moreover, it has been proposed that in many countries there is a wider pending public health crisis related to rising caries prevalence [31] with little sign that inequalities in oral health are narrowing [32].

It has been suggested that in Scotland there have been steady improvements in health over recent decades. However, in spite of this, the ‘inequality-gap’ in health outcomes is generally considered to be getting wider, as the most socioeconomically deprived communities are improving at a slower rate than the most affluent communities [33]. Nevertheless, this Scottish study has recorded substantial improvements in caries indices across the whole socioeconomic spectrum. The influential Marmot Report (2010) [34] considered that change of this nature is necessary if inequalities in health are to decrease. Figures 1 and 2 show that there is a pervasive direct relationship between SES measured by DepCat and both morbidity and prevalence of caries in Scotland. At baseline the odds-ratio for dental caries among Glasgow children was 34% greater than that of their peers from the Rest of Scotland. This is indicative of a marked geographic dental health inequality, over and above the better known socioeconomic inequality in the caries distribution. In the course of the study interval, the excess odds-ratio for Glasgow children decreased substantially until there was no longer any evidence of geographic inequality to the detriment of Glasgow’s children.

Gakidou and King advocate the use of a ‘whole population’ perspective to consider inequalities in health over and above SES inequality, arguing that segmentation by SES in some inequalities measurement methodologies imposes a value judgement [35]. The use of SIC, SIC 10 and SCIM 10 trends have permitted the dispersion of dental caries in the ‘whole population’ of five-year-olds resident in Glasgow and the Rest of Scotland to be considered in this way. The SCIM 10 demonstrates substantial reduction in the whole population dispersion of infants’ caries in Glasgow and the rest of Scotland (68% and 62%, respectively). The SIC and the SIC 10 trends provide reassurance that there have been substantial decreases in caries morbidity across Scotland within the worst affected third and tenth of the caries distribution (irrespective of SES) and that the improvement in overall population dental health statistics is not due solely to improvements among those who would earlier have had only marginal disease experience.

The greater prevalence of caries within the Glasgow population was not unexpected, as this has been reported previously in association with SES [36]. The population within the former NHS Greater Glasgow Health Board area comprises around 20% of Scotland’s total population and there is a high concentration of residents living within the urban core of a large metropolitan area. This contrasts with the smaller cities, towns, villages, rural and semi-rural area found in the remainder of Scotland. Urban–rural differences in five-year-olds’ dental health have been reported [37]. However, more pertinent to this study is the lower socio-economic status of the Glasgow children, as this geographic area contains almost half of the most deprived postcode sectors in Scotland within its boundaries.

Donnelly [12] concludes that there is generally a consensus that existence of the phenomenon of the ‘Glasgow Effect’ is beyond doubt. This study aimed to add to previous literature by investigating the extent to which area-based socio-economic status explained the observed differences between Glasgow and the Rest of Scotland and whether, over and above, there was indeed a ‘Glasgow Effect’ with impact on five year-old childrens’ dental health outcomes. Findings in this study are in contrast to preliminary suggestions by other authors that a ‘Glasgow Effect’ may not extend to the very young [10], as there was clearly a detrimental ‘Glasgow Effect’ evident throughout the 1990s. Children residing in Glasgow had higher overall morbidity and prevalence of dental caries than those living elsewhere in the Rest of Scotland, after controlling for their area-level socio-economic status. This is therefore in keeping with observations of the ‘Glasgow Effect’ in adult populations [5,7,9,10,12,13,38,39] and may provide evidence of this generally detrimental geographic effect at an earlier age than observed previously.

This retrospective study is constrained by the nature of the caries datasets which were available for analyses. It would have been ideal to have had access to individual level SES information for subjects, over and above. However, no such information is held in relation to Scotland’s national caries data sets and the limitations associated with use of an area-based measure of socio-economic status are acknowledged. It is possible that the area-based ‘Glasgow Effect’ demonstrated in the early years of our study could be explained by individual SES measures. Other possible explanations found within previous literature include environment, behaviours, migration, social, cultural, genetic differences or that the Glasgow population has a greater vulnerability [1-3,5-11,39].

The disappearance of the earlier apparent detrimental ‘Glasgow Effect’ on five-year-olds’ dental health began to be mitigated during a period temporally associated with Scotland’s pilot interventions for the *Childsmile* programm*e* [17,18] and other contemporary child health and welfare interventions e.g. *‘Starting Well’* [40]. This continued following ongoing implementation of the *Childsmile* programme across Scotland from 2006 [19,20]. However, due to the ecological nature of the interventions we are cautious about inferring any causality, although we have previously demonstrated a very strong association between the *Childsmile* programme and improved dental health outcomes [21].

Adult studies attempting to fully explain the ‘Glasgow Effect’ found that most excess risk for health outcomes could be explained by area-based and individual level SES, although some remain unexplained [2]. Notwithstanding this, an interesting finding is that while the ‘Glasgow Effect’ was evident in child dental health during the initial years of collection of national data, throughout the first decade of the 21st century, this effect, based on an area-based measure of deprivation, diminished to the point where it is now no longer evident. Observation of the reduction of any ‘additional area-based deprivation effect’ over time is an important addition to the ‘Glasgow Effect’ literature.

## Conclusions

At baseline, we demonstrated a further example of the ‘Glasgow Effect’ in the arena of dental health. This was observed in an age-group which has not featured previously in the Glasgow Effect literature. This Glasgow Effect was mitigated over time until its eventual elimination in 2012, by which point there is no higher level of dental caries in the Glasgow area’s five-year-old population than in the age group in the Rest of Scotland.

## Abbreviations

NDIP: National Dental Inspection Programme
GGHB: NHS Greater Glasgow Health Board
SIC: Significant Caries Index
SIC 10: SIC of the most deprived decile
SCIM 10: Scottish Caries Inequality Metric
SES: Socio-economic status
